# A link between transcription fidelity and pausing *in vivo*

**DOI:** 10.1080/21541264.2016.1274812

**Published:** 2017-01-10

**Authors:** Pamela Gamba, Katherine James, Nikolay Zenkin

**Affiliations:** Centre for Bacterial Cell Biology, Institute for Cell and Molecular Biosciences, Newcastle University, Newcastle upon Tyne, UK

**Keywords:** collisions of transcription with replication, fidelity, Gre, misincorporation, pausing, proofreading, TFIIS, transcription

## Abstract

Pausing by RNA polymerase is a major mechanism that regulates transcription elongation but can cause conflicts with fellow RNA polymerases and other cellular machineries. Here, we summarize our recent finding that misincorporation could be a major source of transcription pausing *in vivo*, and discuss the role of misincorporation-induced pausing.

Elongation of transcripts by DNA-dependent RNA polymerases (RNAP) is a temporally discontinuous process: interactions with certain DNA (or RNA) sequences, DNA lesions, misincorporation events, or encounters with other DNA-bound proteins are able to trigger programmed or accidental transcriptional pausing.

The majority of characterized pauses are events that branch off the main elongation pathway, and are believed to start with an isomerization of the elongation complex (EC) into an *elemental pause* state that is catalytically inactive but does not alter the translocation state of RNAP.^1^ Once this transient rearrangement has occurred, it can be further stabilized by subsequent events that follow distinct pathways, and result in the formation of long-lived pauses. The stabilization can occur by backtracking (when the 3′ end of the RNA disengages from the active site and extrudes into the secondary channel, maintaining the EC in an inactive state), by the formation of a hairpin in the nascent RNA strand, by regulatory proteins, or by the interaction with DNA sequences that contact the RNAP downstream of the active site.^1-3^

Another type of RNAP pause, described by us relatively recently, is caused by slow translocation between the pre-translocated and post-translocated states following NMP incorporation.^4^ Pre-translocated pauses are caused by the sequence of the RNA–DNA hybrid and surrounding DNA,^4,5^ and recent genome-wide studies suggested that they are very frequent (see below).^2,6^ Pre-translocated pauses can be modulated by transcription factors,^7,8^ or by antibiotics, such as tagetitoxin.^9^ Pre-translocated pauses may also be a starting point for long-lived pauses,^4^ or serve as a prerequisite for the elemental pause state.

Pauses that are induced by the DNA sequence are ubiquitous. Single molecule studies suggested, and *in vivo* genome-wide studies recently showed, that pause sites occur with an average frequency of 1 per 100 bp in the *Escherichia coli* genome.^2,6^ Sequence alignments of pause sites also revealed a consensus sequence spanning 16 nt, whose stronger determinants (on the non-template strand of DNA) are G_−10_ at the upstream edge of the RNA:DNA hybrid, and Y_−1_G_+1_, where −1 corresponds to the RNA 3′ end.^2,6^ The two studies suggested that the consensus pause sequence induces pre-translocated pausing, although it remains unclear whether it is further stabilized by backtracking.^2,6^ Another study, however, suggested that pausing at consensus sequences may not be limited to the pre-translocated state.^10^ These pauses appeared to be overrepresented at translation start sites, and enriched within the first 100 nt of expressed genes.^6^

A distinct type of pause is caused when the σ factor fails to disengage from the core enzyme after promoter escape, and recognizes −10-like promoter sequences in the transcribed DNA (usually in the promoter proximal regions), thus causing a pause in elongation.^11-13^ These pauses also appear to be stabilized by backtracking.^12,14^

## Regulatory roles of transcriptional pausing

Specific regulatory roles have been characterized in detail for some individual pauses.^1,15^ Hairpin-stabilized attenuator pauses indirectly couple transcription with translation at many amino acid biosynthetic operons in bacteria, allowing the modulation of their expression in response to changes in nutrient availability.^16^ Halting of RNAP at key positions may also be required for the recruitment of regulators. The bacterial *ops* pause (operon polarity suppressor) allows the EC to bind RfaH, a regulator that suppresses pausing and termination downstream the *ops* site.^17^ Binding of the antiterminator Q of phage lambda requires the σ^70^-dependent promoter-proximal pausing of the RNAP.^11^

Pausing may influence the folding of nascent transcripts, and has been shown to be required for the correct assembly of some biologically active RNAs.^18,19^ A pause signal located between the upstream and downstream portions of long-range helices is needed to guide proper folding of the RNA component of Ribonuclease P from *E. coli*, and of two other conserved non-coding RNAs, probably by preventing the formation of stable non-native structures.^20^ Pausing affects RNA folding or ligand binding of several riboswitches, and therefore affects the regulation of the downstream transcription units.^19^

Pausing is the first step of both intrinsic and Rho-dependent termination,^1^ and may play a regulatory role during transcription initiation: single-molecule fluorescence studies recently revealed a long pause at the *lac* promoter, occurring at the transition from 6- to 7-nt RNA, which is suggested to be conserved in other promoters.^21^

In eukaryotes, RNAPII can pause between the promoter and the first nucleosome, 30–60 nt downstream of the transcription start site.^22^ This promoter-proximal pausing depends on different factors, and the escape into productive elongation is often (i.e., for half of the active *Drosophila* and mammalian genes) rate limiting, and highly regulated, thus playing a critical role in regulating metazoan gene transcription.^23^ Pausing by RNAPII may also facilitate polyadenylation or contribute to splice site selection in alternatively spliced mRNAs.^1,24^

Besides the specific roles of elongation pauses in regulating the expression of specific genes, pauses are thought to be required to reduce the pace of transcription elongation, and therefore allow for the coordination of transcription with translation in bacteria.^1^

## Deleterious effects of transcriptional pausing

Bacterial RNAP and the replisome move simultaneously along the same template, but the former is 12–30-fold slower than the latter.^25^ Therefore, conflicts between the two machineries are inevitable, and especially frequent in actively growing cells and in heavily transcribed regions. The outcomes of such events depend on the orientation of the collisions and on the translocation state of the EC. Co-directional collisions with actively transcribing ECs do not impede replisome progression *in vivo*, and *in vitro* studies showed that the replisome can readily dislocate the RNAP and use the mRNA as a primer to continue replicating the leading strand.^26^ Head-on collisions with transcribing RNAP are more severe and have been shown to arrest the replication forks both *in vivo* and *in vitro*.^27^ Accordingly, highly-expressed, long, and essential genes are preferentially located on the leading strand in bacterial genomes,^15^ and genetic instability following head-on collisions was reported.^28^ In the presence of backtracked ECs, co-directional collisions are particularly detrimental and were shown to cause double-strand breaks (DSBs) *in vivo*.^29^ A recent study in *Streptococcus pneumoniae* suggested that trailing RNAPs might queue behind the stalled one, thereby forming RNAP “traffic jams,” having detrimental consequences on gene expression and forming much more potent obstacles for the replication machinery.^30^

Different factors have been shown to play a role in removing, reactivating, or preventing backtracked ECs (and/or RNAP queues caused by them), with their effects normally becoming apparent only when one of the other pathways is disrupted.^15^ The elongation factors GreA and GreB (in *E. coli*; many bacteria have only one Gre factor) can reactivate backtracked ECs by promoting a transcript cleavage reaction at the active site that generates a new 3′ end.^31^ Mfd is a translocase that can bind upstream of a stalled EC and can either rescue transcription or remove the complex by driving forward translocation, and is a key factor in transcription-coupled repair of DNA lesions in *E. coli*.^32^ Arrested ECs can also be released by Rho-dependent termination.^33^ DksA binds the RNAP in the secondary channel and was shown to prevent transcription arrest upon ribosome stalling under amino acid starvation.^34^ Trailing RNAPs themselves were suggested to cooperate during elongation to prevent backtracking of a leading RNAP,^35^ although recent results rather supported an alternative “traffic jams” model, as mentioned above.^30^

## Misincorporation induces pausing

**Figure 1.**
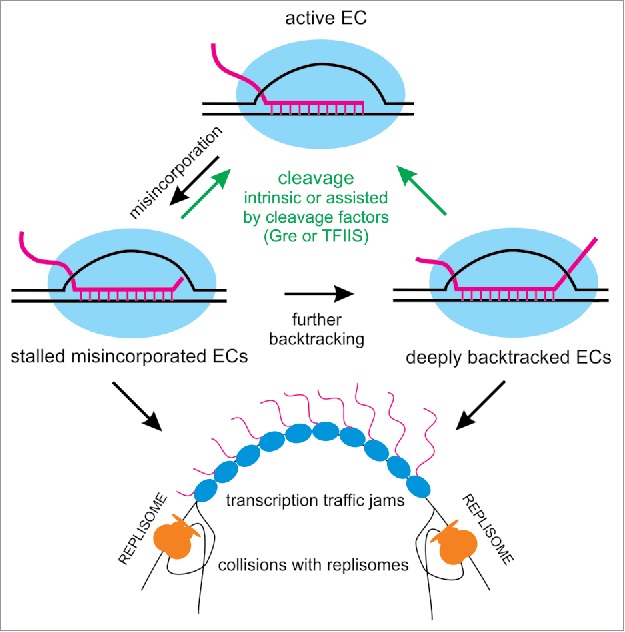
Misincorporation-induced pausing. Upon misincorporation, the elongation complex (EC; RNA is magenta, RNAP is blue, DNA is black) is stabilized in a 1 base pair backtracked state, possibly through a frayed intermediate,^54^ which may then lead to further backtracking. Misincorporated and deeply backtracked ECs result in long-living pauses of transcription, until resolved by intrinsic or factor-dependent cleavage. These paused ECs may cause RNAP traffic jams and collisions with replication (replisomes replicating leading and lagging strands are shown in orange).

Misincorporation events cause long-lived pausing *in vitro* because they induce backtracking of the EC by 1 bp (Fig. 1).^36,37^ Transcription can resume upon the hydrolysis of the second phosphodiester bond of the transcript by the active site of the RNAP, which removes the last two nucleotides.^37^ *In vitro*, this reaction is greatly stimulated by the cleavage factors Gre in bacteria, and TFIIS for the eukaryotic RNAPII.^31,38^

Until recently, misincorporation-induced pauses could not be investigated *in vivo* because they occur randomly and are transient. However, based on the small effects that the absence of cleavage factors has on the overall error rate of RNAP, they were thought to be very rare and, therefore, to have negligible effects on the overall pausing of transcription.^30,39^

## Misincorporation is a major source of transcriptional pausing *in vivo*

The development of Native Elongating Transcript sequencing (NET-seq) has made it possible to selectively sequence transcripts that are bound to transcribing RNAP, and, therefore, to investigate genome-wide pausing *in vivo* in different model organisms.^2,6,24,40^ Importantly, NET-seq is based on the sequencing of the 3′ ends of nascent transcripts and, therefore, reports on the exact position of the ECs and can also be used to visualize misincorporation events.

Recently, we analyzed transcriptional errors in earlier NET-seq data from *E. coli* and *S. cerevisiae*, for both wild-type and cleavage factor deficient mutants.^6,40^ We found that all strains carried an unexpectedly high proportion of misincorporations at the 3′ end position, which we quantified as 3% and 1% of all ECs in wild-type *E. coli* and *S. cerevisiae*, respectively.^41^ In the mutant strains lacking cleavage factors, the misincorporated ECs were ∼5% and 7%, respectively.^41^ These values are considerably higher than expected from the overall error rate of RNAP, 10^−3^–10^−6^.^42^ A somewhat lower proportion (0.1% and 1% for wild-type and mutant *E. coli* strains) of misincorporated ECs was reported by a different study,^10^ and we have discussed the possible cause of such differences elsewhere.^41^ Given that misincorporation causes stable backtracking, it is likely that these higher proportions are mainly caused by the accumulation of misincorporated complexes due to their slow resolution, even in wild-type cells. Importantly, the misincorporated complexes are non-productive in the formation of a mature RNA until they are resolved, therefore, such accumulation would not affect the error rate of final RNA products. This explains why this high proportion of misincorporation complexes might have been overlooked.

**Figure 2.**
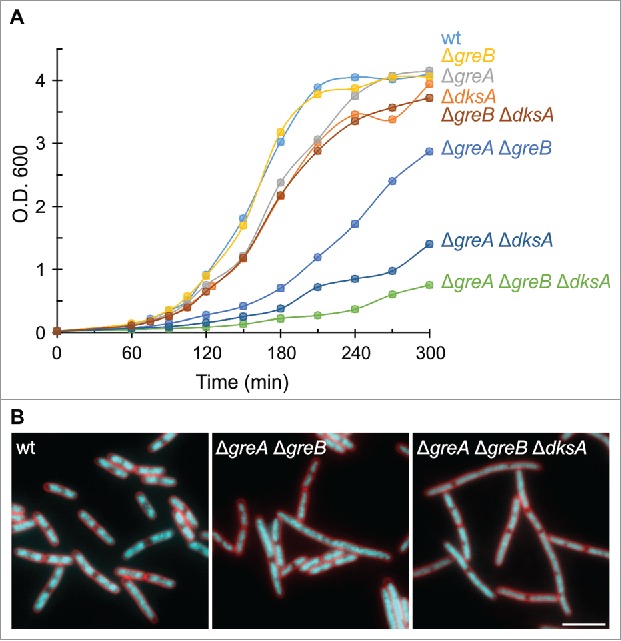
Growth and morphological defects caused by deletion of *greA, greB*, or *dksA* in *E. coli*. (A) Growth curves of single, double, and triple mutants in EZ Rich Defined medium at 37°C. All strains are derivative of the wild-type strain MG1655. Scarless deletions of the whole coding sequence of *dksA* and *greA* genes, and the first 318 bp for *greB* (in order not to delete a putative promoter for the downstream gene *yhgF*), were performed using standard protocols for λ Red-mediated recombination and P1 transduction (construction details will be published elsewhere). At least three replicates were performed and a representative experiment is shown for each strain. (B) Microscopic analysis of wild type, Δ*greA* Δ*greB* and Δ*greA* Δ*greB* Δ*dksA* strains grown in EZ medium at 37°C and imaged at an O.D.600 of 1.2, 1.3, and 0.2, respectively. Fields highlighting cells with morphological defects are shown as an overlay of FM5–95 (red) and DAPI (cyan) channels. Cells were mounted on microscope slides coated with a thin layer of 1.2% agarose. Images were acquired with a Nikon Eclipse Ti microscope, equipped with a Sony Cool-Snap HQ2 cooled CCD camera, and using Metamorph imaging software (Universal Imaging). Scale bar: 5 µm.

These results imply that misincorporation could be a major cause of backtracked pausing *in vivo*, and, consequently, a significant source of conflicts with the replication machinery and other trailing RNAPs both in bacteria and eukaryotes (Fig. 1). Whether the ubiquitous sequence-dependent pauses involve backtracking is controversial, since deletion of *gre* genes did not alter significantly the pattern of pausing in a first study in *E. coli*,^6^ but seemed to do so in a later study.^10^ However, misincorporated ECs are stabilized in the backtracked state and must be a subject for factor-assisted cleavage. Therefore, it appears possible that the most significant contribution of Gre factors *in vivo* is the reactivation of these stalled complexes, which reduces conflicts with the replisome and trailing RNAPs, rather than the improvement of transcriptional fidelity itself. DksA might contribute to the same cause by decreasing misincorporation events.^43,44^ Consistently, *E. coli* double mutants lacking DksA and either of the Gre factors show a significantly reduced growth rate even in nutrient rich conditions, and these growth defects become extremely severe in the triple mutant (Fig. 2A). We also observed a significant degree of filamentation and diffuse nucleoid morphology in the triple mutant (Fig. 2B), which may point to defects in replication or chromosome segregation. Decrease of replication fork progression was in fact observed in the triple mutant, and the three factors are known to prevent replication arrest during nutrient stress.^45^ Furthermore, we observed similar morphological defects in a Δ*greA* mutant of *S. pneumoniae*, which does not have other secondary channel binding factors.^30^ Of course, the severe growth phenotypes could also be due to the accumulation of distinct defects, and direct evidence is still missing.

The viability of even the triple Δ*greA* Δ*greB* Δ*dksA* mutant suggests that *E. coli* cells can still cope with the misincorporated ECs. It is possible that other factors, such as Mfd or Rho, which can displace paused ECs from the template, may also contribute in overcoming the misincorporated ECs. In addition, the replisome itself may be able to displace some of the complexes, since most of them will be oriented co-directionally, and use the transcript as a primer.^26^ The misincorporated NMP at the 3′ end of the RNA primers may be a problem for its extension by DNA polymerase, which, however, could be rectified by RNase H or 3′ exonucleases (such as the 3′ exonuclease activity of DNA polymerase) by generating a correct 3′ end of the primer. Alternatively, the resolution of the misincorporated ECs in the absence of cleavage factors could be helped by intrinsic transcript-assisted proofreading activity of the RNAP active center.^37^ Similar mechanisms are likely involved also in eukaryotes.

Interestingly, analysis of the hotspots of misincorporated ECs in *E. coli* revealed that these hotspots are far less abundant in protein-coding sequences compared with transcribed untranslated regions (1.34 and 10.68 hotspots per 0.1 Mb, respectively), while no such bias was seen in *S. cerevisiae*.^41^ Bacteria might therefore have minimized sequences causing frequent misincorporation events or evolved mechanisms for reducing or correcting misincorporation at hotspots specifically in protein coding regions. Intriguingly, since coupled ribosomes were shown to control elongation and pausing by RNAP,^46,47^ translation itself might suppress misincorporation events or, more likely, promote extension of the misincorporated RNA.

The pattern of 3′ misincorporations that we observed had a strong bias toward G>A (misincorporation of A instead of G), which is consistent with previous observations.^10^ This earlier study suggested that, in sequence-dependent pausing, a C_−1_G_+1_ motifs (non-template DNA sequence) increase the rate of G>A misincorporation at +1 position.^10^ Indeed, at G>A hotspots, we also observed a clear bias for C preceding the misincorporated 3′ end, however, we did not see strong pausing at these C_−1_ positions.^41^ Therefore, misincorporations might occur at those positions without the involvement of a pause. Importantly, most of the misincorporation events happened away from hotspots, and the sequence bias toward C_−1_ for all G>A misincorporations was, in comparison to hotspots, lower in *E. coli* and non-present in *S. cerevisiae*, suggesting that G>A misincorporation is a quite random event.^41^

Misincorporation-induced backtracked pauses may interfere with the progression of replication forks and significantly alter the gene expression patterns, by themselves or by causing RNAP jams.^30^ It appears likely, therefore, that some phenotypes found in human diseases,^48-51^ splicing defects,^52^ or epigenetically inheritable changes in gene expression,^53^ which were all linked to transcription infidelity, are in fact caused by the deleterious effects of stalled misincorporated complexes rather than by the correctness of the final RNA products.
